# Development of Japanese and Indonesian Versions of the electronic-Health Literacy Scale

**DOI:** 10.31662/jmaj.2024-0282

**Published:** 2025-08-08

**Authors:** Yayoi Shoji, Andi Masyitha Irwan, Ryota Ochiai, Syahrul Syahrul, Eriko Shinohara, Andi Muhammad Fiqri, Shoko Takeuchi, Erfina Erfina, Mariko Iida, Ariyanti Saleh, Fusae Moriguchi, Sachiyo Nakamura, Yuka Kanoya

**Affiliations:** 1 Public Health Unit, Global Cooperation Institute for Sustainable Cities, Yokohama City University, Yokohama, Japan; 2 Department of Nursing, Graduate School of Medicine, Yokohama City University, Yokohama, Japan; 3 Faculty of Nursing, Hasanuddin University, Makassar, Indonesia; 4 Graduate School of Health Care and Nursing, Juntendo University, Urayasu, Japan

**Keywords:** e-Health literacy, health behavior, scale development

## Abstract

**Introduction:**

Reportedly, electronic-Health (eHealth) literacy is lower in younger people than in those in their 40s and 50s, despite the Internet being an essential information source for the youth. We developed Japanese and Indonesian versions of the e-Health Literacy Scale (e-HLS).

**Methods:**

Conceptual equivalence was verified using the forward-backward translation method. First- to fourth-year nursing students at two universities in Japan and Indonesia were surveyed using a web-based questionnaire, including a 12-item e-HLS. Data analysis involved item analysis, factor analysis, and validation of convergent and discriminant validity, reliability, discriminative validity, and cross-cultural validity. The study was approved by the Indonesian and Japanese ethics boards.

**Results:**

Ninety-nine Japanese and 407 Indonesian participants responded to the survey (response rate: Japan 23.9%, Indonesia 89.6％). Neither country showed item distribution skewness, and no ceiling or floor effects were detected. Confirmatory factor analysis supported the same three-factor structure as the original e-HLS. Discriminative validity indicated that fourth-year students had higher overall, functional, and interactive health literacy scores than first- to third-year students. There were no significant differences between the two groups in terms of critical health literacy. Cronbach’s alpha coefficients were 0.86 overall and 0.74-0.83 for each of the three domains. Regarding cross-cultural validity, configural invariance and full metric invariance models showed a good fit, but scalar invariance was not supported.

**Conclusions:**

From the results, the content validity, construct validity (structural, cross-cultural, convergent, and discriminant validity), and reliability (internal consistency) of the Japanese and Indonesian versions of the e-HLS were confirmed.

## Introduction

Health literacy is defined as individuals’ ability to find, understand, and use information and services that can help them make decisions and take actions related to their health and that of others ^(1)^. Nutbeam ^(2)^ categorized health literacy into functional skills, which involve reading and understanding information; interactive skills, which involve communication and social interaction; and critical skills, which involve critical analysis of information. A meta-analysis conducted in 2011 reported that low health literacy was associated with poorer health behaviors, including increased hospitalization, greater use of emergency care, and poorer ability to demonstrate taking medications appropriately ^(3)^. In recent years, within the domain of health literacy, the concept of eHealth literacy, which refers to the ability to seek, find, understand, evaluate, and apply health-related information from electronic sources to address health issues, has gained attention ^(4)^. As of 2015, among the World Health Organization member states, two-thirds of the countries indicated the importance of eHealth literacy in the healthcare field ^(5)^.

The International Telecommunication Union, the specialized agency of the United Nations for information and communication technologies, reported that in 2020, 71% of the world’s youth aged 15-24 years would be using the Internet, making them 1.24 times more likely to be connected compared to 57% of other age groups. Young people are reported to have a higher likelihood of Internet connectivity than other age demographics ^(6)^. Research on college students’ eHealth literacy indicated that it involves functional skills, such as effectively utilizing the Internet for understanding information; interactive skills, encompassing effective communication and social interaction; and critical skills, including the ability to discern and evaluate information quality ^(7)^. Students with higher critical eHealth literacy were found to be more capable of effectively using health care services ^(7)^, and higher levels of critical eHealth literacy were reported to promote students’ health behavior practices ^(8)^. Furthermore, age and eHealth literacy were found to be related ^(9)^; studies indicated that eHealth literacy among 20-year-olds was significantly lower than that among 40- and 50-year-olds ^(10)^, and that eHealth literacy was not necessarily high compared to the rate of Internet use. Several studies have focused on eHealth literacy among college students ^(11), (12), (13)^. While Internet use among young people is high, their eHealth literacy is not necessarily high in terms of correctly searching for and analyzing health information, indicating the need for improvement of eHealth literacy among young people.

Research on eHealth literacy has steadily increased from 2011 to 2016 ^(9)^. In addition to this research trend, the global coronavirus disease 2019 (COVID-19) pandemic may have contributed to the increase in literature related to electronic-Health (eHealth) literacy owing to the decline in face-to-face communication and the surge in electronic sources of information. A systematic review aimed at assessing the role of eHealth literacy in preventive health behaviors associated with COVID-19 found that eHealth literacy had a significant impact on awareness, knowledge, and adherence to health behaviors during the COVID-19 pandemic and contributed to increasing public awareness through the media to prevent and reduce COVID-19 incidence ^(14)^. This study showed that eHealth literacy was a crucial component of health literacy in advancing preventive strategies related to COVID-19 measures. Based on this, it is important to enhance eHealth literacy in preparation for future outbreaks of new infectious diseases.

The e-Health Literacy Scale (e-HLS) was developed by Chiang et al. ^(15)^ to assess eHealth literacy among young individuals. In a pilot survey involving 148 Taiwanese university students, exploratory factor analysis revealed a three-factor structure consistent with Nutbeam’s ^(2)^ eHealth literacy components ^(15)^. A subsequent study involving 455 participants confirmed the reliability and validity of the scale through confirmatory factor analysis (CFA) ^(15)^. Among various instruments to measure eHealth literacy, including the Digital Health Literacy Instrument ^(16)^, The eHealth Literacy Scale (eHEALS) ^(17)^, e-Health Literacy Assessment Toolkit ^(18)^, and Transactional eHealth Literacy Instrument ^(19)^, the e-HLS has its advantages in that it is youth-specific and consists of the three-factor structure consistent with Nutbeam’s components. Furthermore, its availability in multiple languages allows international comparisons. Hence, this study aimed to develop Japanese and Indonesian versions of the e-HLS, validate their reliability and validity, and assess their intercultural validity, thereby maximizing their potential for international comparisons.

## Materials and Methods

### Development of Japanese and Indonesian versions of the e-HLS

The Japanese and Indonesian versions of the e-HLS were developed in accordance with the international standards for scale development and the COnsensus-based Standards for the Selection of Health Measurement Instruments (COSMIN) ^(20)^. Permission for scale development and consent for translating the original e-HLS were obtained from Chia-Hsun Chiang ^(15)^.

For the Japanese version, two researchers including the authors performed forward translation, developing a provisional version. An English back-translation was performed by a specialist not involved in the forward translation, ensuring conceptual equivalence. Similarly, for the Indonesian version, four Indonesian researchers conducted a forward translation, resulting in a provisional version. An Indonesian researcher who had not participated in the forward translation performed back-translation, which was then reviewed by the four authors to ensure conceptual equivalence with the original scale. Additionally, considering religious and cultural aspects, researchers from each country reviewed the conceptual and semantic aspects of the translated questionnaire items.

Next, content validity, comprehensiveness, and comprehensibility were evaluated according to the COSMIN criteria. For appropriateness, the original author reviewed the Japanese and Indonesian back-translated versions of the e-HLS to ensure accuracy. Comprehensiveness was not the focus of this study, as the priority was alignment with the original scale without adding new items. Comprehensibility was evaluated in Japan and Indonesia through an anonymous, web-based questionnaire survey involving four predoctoral nursing students. In addition to the 12 e-HLS items, participants provided free-text responses based on COSMIN’s comprehensibility criteria. The feedback, such as the need for estimated time per question and occasional difficulty in understanding certain items, was reviewed. However, the responses indicated that no questions were particularly difficult to answer. After re-evaluating the scale among researchers, no modifications were determined to be necessary, resulting in the final Japanese and Indonesian versions of the e-HLS.

### Psychometric evaluation of the Japanese and Indonesian e-HLS

#### Study design

This study employed a cross-sectional observational design using anonymous self-administered questionnaires. The survey period for the Japanese version was from March 1, 2022 to April 19, 2022, whereas that for the Indonesian version was from January 13, 2023 to February 1, 2023. Eligible participants received web-based questionnaires and were invited to participate. The participants were instructed to respond individually without seeking input from others or using external references. Data were collected online.

#### Participants

Eligible participants were students enrolled in nursing programs at Yokohama City University Medical School (Japan) and Hasanuddin University (Indonesia), who provided consent to participate in the study. Exclusion criteria encompassed students who were on leave from the university during the study period or had difficulty reading or writing in the survey language. Nursing programs were chosen as the focus of this study because of the similarity of the 4-year programs at both universities, which allowed for enhanced comparability. The target sample size aimed to follow COSMIN’s recommended number of participants ^(21)^, setting a target of 100 participants based on the “excellent” category (number of items × 7 and at least 100 participants). A conservative response rate of 25% was estimated for this study. The questionnaires were distributed to 413 and 454 students in Japan and Indonesia, respectively, totaling 867 participants.

#### Survey content

The primary survey items consisted of the Japanese or Indonesian version of the e-HLS. Both versions comprised 12 items with three subscales: functional (3 items), interactive (4 items), and critical health literacy (5 items). Responses were rated on a 5-point Likert scale from “1 = Strongly Disagree” to “5 = Strongly Agree.” The scores were calculated by summing the points assigned to each response option, with higher scores indicating higher eHealth literacy. Additional demographic information included year of study, age, sex, living arrangements, restrictions owing to cultural or religious reasons, and health status.

#### Analysis

The analysis included all participants who completed the questionnaires. Statistical analyses included calculation of descriptive statistics; confirmation of item statistics, construct validity, convergent and discriminant validity, reliability; discriminative validity; and multi-population concurrent analysis.

#### Descriptive statistics

Descriptive statistics produced the proportion of participants’ sex, age, year of study, prohibited items, underlying diseases, residential type, and body mass index. Mean scores and standard deviations for each e-HLS item were also calculated.

#### Item analysis

Item analysis was used to identify the ceiling and/or floor effect for each item. A ceiling effect was defined as a mean plus standard deviation of five or more points, the maximum possible value for which a score could be taken; a floor effect was a mean minus standard deviation of one point or less, the minimum possible value for which a score could be taken.

#### Factor analysis

To confirm construct validity, a CFA was first conducted to assess the level of model fit. The criteria for the CFA indicators were Goodness of Fit Index (GFI), Adjusted Goodness of Fit Index (AGFI), Comparative Fit Index (CFI) ≥ 0.90, and Root Mean Square Error of Approximation (RMSEA) < 0.08. Exploratory factor analysis (EFA) was not conducted in this study because the e-HLS is an existing validated scale with a well-established factor structure. The original scale has undergone rigorous validation. Furthermore, our adaptation process followed standard translation and cultural adaptation procedures, including forward and backward translation and expert review, to ensure conceptual equivalence. Therefore, CFA was employed to evaluate the fit of the predefined factor structure.

#### Convergent and discriminant validity

Convergent and discriminant validity were calculated from the correlation coefficients between each item score and each of the two attributed and unattributed subscales. The scaling success rate for each subscale was determined from the frequency with which the convergent correlation was higher than the discriminant correlation.

#### Internal consistency

To examine the reliability, Cronbach’s alpha coefficient and item-total (I-T) correlation analysis (p < 0.2) were performed for the entire scale and each factor to check for internal consistency. I-T correlation analysis was used to check the correlation between the score of each item and the subscale to which the item belonged, and items with p < 0.2 were not included in the adopted items. A Cronbach’s α reliability coefficient exceeding 0.7 was considered satisfactory ^(22)^, and values below 0.5 were considered unsatisfactory.

#### Discriminative validity

Previous studies indicated that the eHealth literacy of fourth-year university students was significantly higher compared with students in other years ^(23)^. Using data from both countries, an independent *t* test was conducted between two groups, one consisting of first- to third-year students (397 participants) and the other of fourth-year students (109 participants), to determine whether eHealth literacy increased as students progressed to higher academic years.

#### Cross-cultural validity

The cultures and religions of Japan and Indonesia are different, and the factor structures of their respective e-HLS may differ as well. Using data from the two countries, multigroup CFA (MGCFA) was conducted using three models: an unconstrained model, a metric model in which factor loadings were considered equal, and a scalar model in which factor correlations were also considered equal. The results of the MGCFA were interpreted in terms of which model fit best by adding stepwise constraints to the model. The goodness of fit of each model was evaluated using RMSEA, CFI, and the Tucker-Levis Index.

IBM SPSS 28.0.1.0 (142) was used for simple tabulation, examination of internal consistency, and discriminative validity (all statistical significance levels were set at p < 0.05), and Amos 29 was used for CFA and MGCFA.

### Ethical considerations

This study was approved by the Ethics Committees of Yokohama City University (approval number: A210100029, approval date: February 2, 2021) and Hasanuddin University (approval number: UH23010022, approval date: January 11, 2023). The research cooperation request documents provided a detailed explanation of the web-based survey’s purpose, time requirements, and ethical considerations. Participants gave informed consent digitally through the web-based questionnaire.

## Results

### Participant characteristics

#### Japan

Valid responses were received from 99 participants (response rate: 23.9%; Table 1), of whom 99% were female and 1% male. Furthermore, 57.6% of the participants were aged 20-21 years, followed by those aged 18-19 and 22-23 years (19.2% each). Regarding year of study, there were 20 first-year students (20.2%), 21 second-year (21.2%), 38 third-year (38.4%), and 20 fourth-year students (20.2%).

**Table 1.** Characteristics of Study Participants.

			N ＝ 506
	Item	Japan *n* = 99	Indonesia *n* = 407
		*n* (％)	*n* (％)
Gender	Female	98 (99.0%)	373 (91.6%)
	Male	1 (1.0%)	34 (8.4%)
Year of Study	First year	20 (20.2%)	106 (26.0%)
	Second year	21 (21.2%)	113 (27.8%)
	Third year	38 (38.4%)	99 (24.3%)
	Fourth year	20 (20.2%)	89 (21.9%)
Age	17 years old	0	5 (1.2%)
	18-19 years old	19 (19.2%)	176 (43.2%)
	20-21 years old	57 (57.6%)	199 (48.9%)
	22-23 years old	19 (19.2)	27 (6.6%)
	24 years old and over	4 (4.0%)	0
Religious restrictions	Yes	4 (4.0%)	345 (84.8%)
	No	95 (96.0%)	62 (15.2%)
Health status	With Underlying disease(s)	12 (12.1%)	57 (14.0%)
	No underlying disease	87 (87.9%)	350 (86.0%)
Living arrangements	Living with parents	70 (70.7%)	165 (40.5%)
	Not living with parents	29 (29.3%)	242 (59.5%)
Body Mass Index	Underweight/Normal weight	97 (98.0%)	355 (87.2%)
	Obese 1-4	2 (2.0%)	52 (12.8%)

#### Indonesia

Valid responses were obtained from 407 participants (response rate: 89.6%), of whom 91.6% were female and 8.4% male. Furthermore, 48.9% of the participants were aged 20-21 years, followed by those aged 18-19 (43.2%) and 22-23 years (6.6%). Regarding year of study, 106 students were in their first year (26%), 113 in the second year (27.8%), 99 in the third year (24.3%), and 89 in the fourth year (21.9%).

### Item analysis

The results showed that no items had ceiling or floor effects in both the Japanese and Indonesian versions (Table 2).

**Table 2.** Item Analysis of the Japanese and Indonesian Versions of the electronic-Health Literacy Scale.

N ＝ 506													
	Japan *n* = 99						Indonesia *n* = 407						I-T Correlation
	mean	SD	Ceiling effect	Floor effect	Percentage of Ceiling effect	Percentage of Floor effect	mean	SD	Ceiling effect	Floor effect	Percentage of Ceiling effect	Percentage of Floor effect	
1 I cannot understand the symbols (such as BMI, Body Mass Index) and wording about health information.＊	4.09	0.69	4.78	3.40	2.0	26.3	4.12	0.79	4.91	3.33	3.4	34.4	0.81
2 I find the online health information difficult to understand.＊	4.03	0.80	4.83	3.23	1.0	25.3	3.97	0.67	4.64	3.30	2.9	17.7	0.77
3 I find the mathematical formulas provided in online health information difficult to calculate. (e.g., the algorithm of calorie consumption, BMI).＊	4.01	0.89	4.90	3.12	2.0	30.3	3.70	0.81	4.51	2.89	0.2	14.5	0.85
4 I can locate health information efficiently through search engines.	3.65	0.98	4.63	2.67	2.0	21.2	3.94	0.62	4.57	3.32	1.5	15.2	0.80
5 I pay attention to and obtain new knowledge about online health information.	3.46	0.97	4.43	2.49	1.0	19.2	4.02	0.58	4.60	3.45	0.2	16.5	0.78
6 I know how to get what I need from online health information.	3.71	0.94	4.65	2.77	1.0	22.2	4.11	0.59	4.70	3.53	0.2	22.6	0.81
7 I understand the online health information I have obtained.	3.98	0.74	4.72	3.24	28.3	26.3	3.98	0.56	4.55	3.42	0.2	13.8	0.76
8 I will think about whether the online health information applies to my situation.	3.95	0.85	4.80	3.10	5.1	28.3	3.89	0.61	4.49	3.28	0.2	12.3	0.67
9 I try to find different sources to verify the credibility of health information.	3.87	1.04	4.91	2.83	2.0	32.3	4.07	0.59	4.66	3.48	0.2	19.9	0.79
10 I evaluate the validity and reliability of online health information.	3.98	0.87	4.85	3.11	1.0	30.3	3.69	0.64	4.33	3.05	0.2	7.1	0.69
11 I will browse various discussions and make a decision or action that is good for health.	3.63	1.04	4.67	2.59	2.0	24.2	3.74	0.60	4.34	3.14	0.2	6.1	0.75
12 When I have questions or doubts about online health information, I use other channels to verify the information.	3.73	0.92	4.65	2.81	11.1	21.2	4.01	0.59	4.61	3.42	0.2	16.7	0.69

＊Reversal items

Based on the original factor structure, I-T correlation analyses were performed, revealing Spearman’s correlation coefficients (*r*) ranging from 0.69 to 0.85 (all p < 0.001) for the correlations between the summed scores of each subscale and individual items. The I-T correlations fell within the recommended range of 0.3 to 0.7, suggesting acceptable internal consistency (Table 2).

### Factor analysis

**Figure 1.**
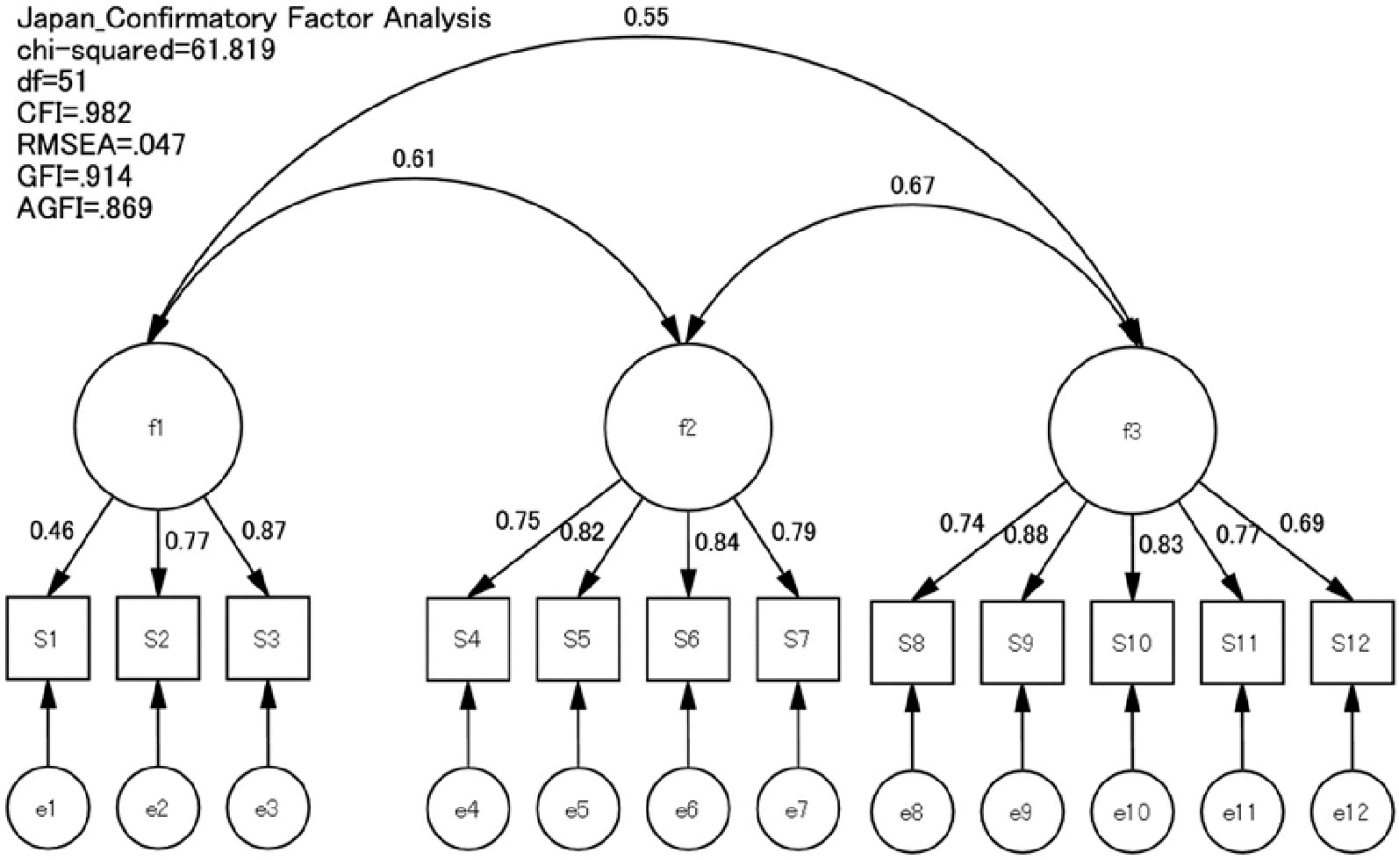
Confirmatory factor analysis of the Japanese version of the electronic-Health Literacy Scale (e-HLS).

**Figure 2.**
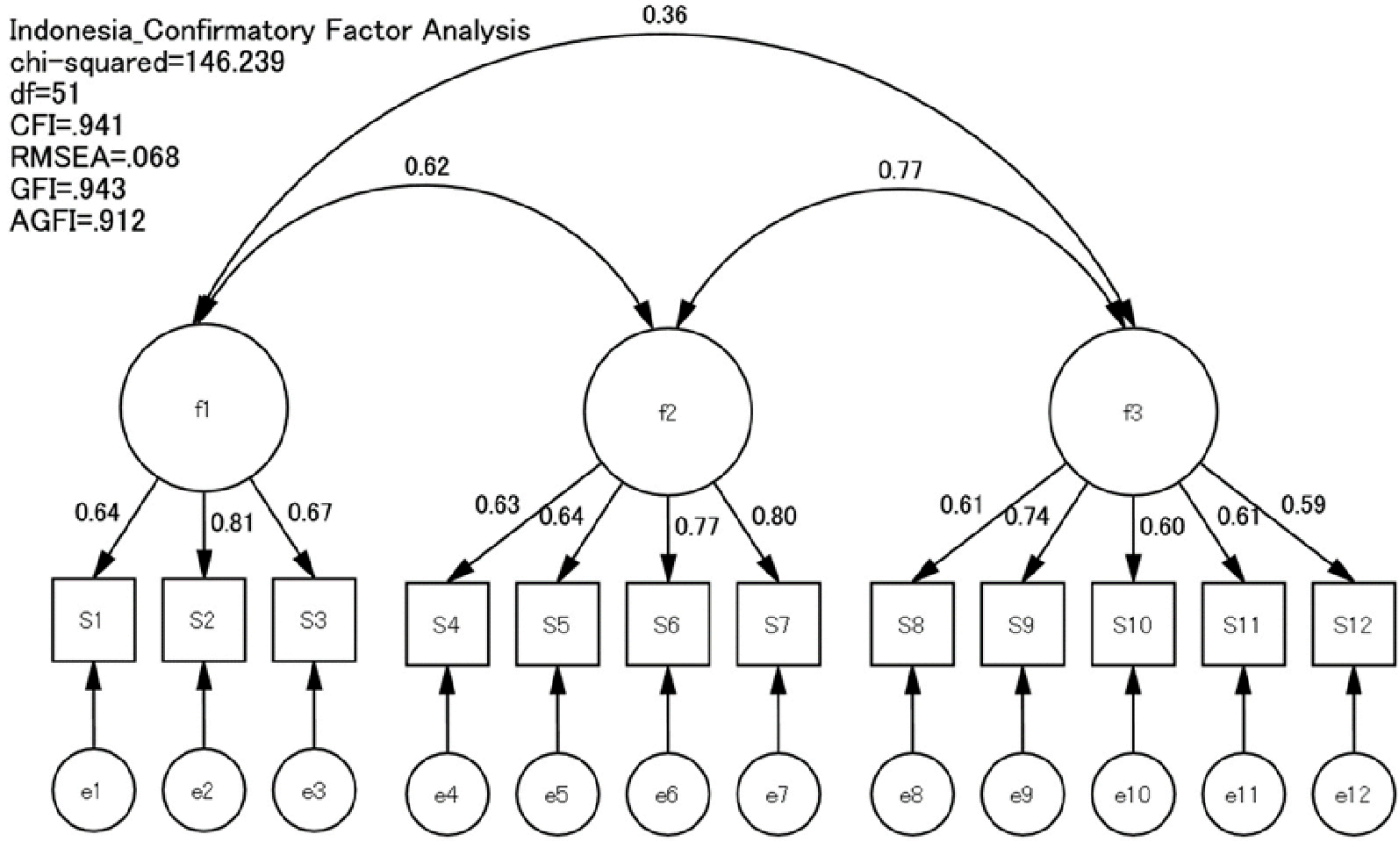
Confirmatory factor analysis of the Indonesian version of the electronic-Health Literacy Scale (e-HLS).

CFA was conducted using data from both countries. The fit indices for the model were as follows: GFI = 0.914, AGFI = 0.869, CFI = 0.982, RMSEA = 0.047, and chi-square = 61.819 (Figure 1 and 2). The first factor, comprising items 1-3, addressed understanding information and was labeled “Functional Literacy.” The second factor, comprising items 4-7, focused on access to information and was termed “Interactive Literacy.” The third factor, comprising items 8-12, evaluated information quality and was named “Critical Literacy.” Thus, the factorial validity of the 12-item Japanese and Indonesian versions of the e-HLS was considered satisfactory.

### Convergent and discriminant validity

The convergent validity coefficients ranged from 0.52 to 0.61 for the functional literacy subscale, 0.63 to 0.72 for the interactive literacy subscale, and 0.54 to 0.71 for the critical literacy subscale. The discriminant validity coefficients ranged from 0.18 to 0.43, 0.35 to 0.49, and 0.24 to 0.54 for the functional, interactive, and critical literacy subscales, respectively. Based on the correlations between the item scales, the assessment revealed a 100% success rate for all three subscales (Table 3).

**Table 3.** Convergent Validity, Discriminant Validity, and Internal Consistency of the Japanese and Indonesian Versions of the electronic-Health Literacy Scale (e-HLS).

Subscale	Item	Convergent Validity	Discriminative validity (Pearson's)			Scaling Success Rate	α
		I-T correlation	Functional	Intractive	Critical		
Functional	1 I cannot understand the symbols (such as BMI, Body Mass Index) and wording about health information.＊	0.52		0.37	0.24	100%	0.74
	2 I find the online health information difficult to understand. ＊	0.61		0.49	0.35		
	3 I find the mathematical formulas provided in online health information difficult to calculate. (e.g., the algorithm of calorie consumption, BMI).＊	0.58		0.36	0.25		
Intractive	4 I can locate health information efficiently through search engines.	0.63	0.40		0.40	100%	0.83
	5 I pay attention to and obtain new knowledge about online health information.	0.66	0.27		0.44		
	6 I know how to get what I need from online health information.	0.72	0.41		0.46		
	7 I understand the online health information I have obtained.	0.65	0.43		0.54		
Critical	8 I will think about whether the online health information applies to my situation.	0.54	0.24	0.45		100%	0.81
	9 I try to find different sources to verify the credibility of health information.	0.71	0.30	0.45			
	10 I evaluate the validity and reliability of online health information.	0.56	0.32	0.36			
	11 I will browse various discussions and make a decision or action that is good for health.	0.63	0.18	0.35			
	12 When I have questions or doubts about online health information, I use other channels to verify the information.	0.56	0.20	0.40			
	alpha coefficient for the overall scale						0.86

＊Reversal items

### Reliability

Regarding internal consistency, the Cronbach’s alpha values for each subscale were as follows: functional literacy = 0.74, interactive literacy = 0.83, and critical literacy = 0.81. The alpha coefficient for the overall scale was 0.86 (Table 3).

### Discriminative validity

As the students’ year of study progressed, the scores for functional and interactive literacy increased. However, critical literacy showed no significant increase with advancement in the academic year. The results of discriminative validity are shown in Table 4.

**Table 4.** Results of Discriminative Validity.

	1st-3rd Year (*n* = 397)		4th Year (*n* = 109)		*pValue*	Cohen's d
	Mean	SD	Mean	SD		
e-HLS Total Score	3.87	0.44	4.09	0.42	<.001	0.44
Functional	3.85	0.60	4.32	0.56	<.001	0.59
Intractive	3.91	0.56	4.10	0.50	0.002	0.55
Critical	3.85	0.53	3.96	0.47	0.051	0.52

### Cross-cultural validity

**Figure 3.**
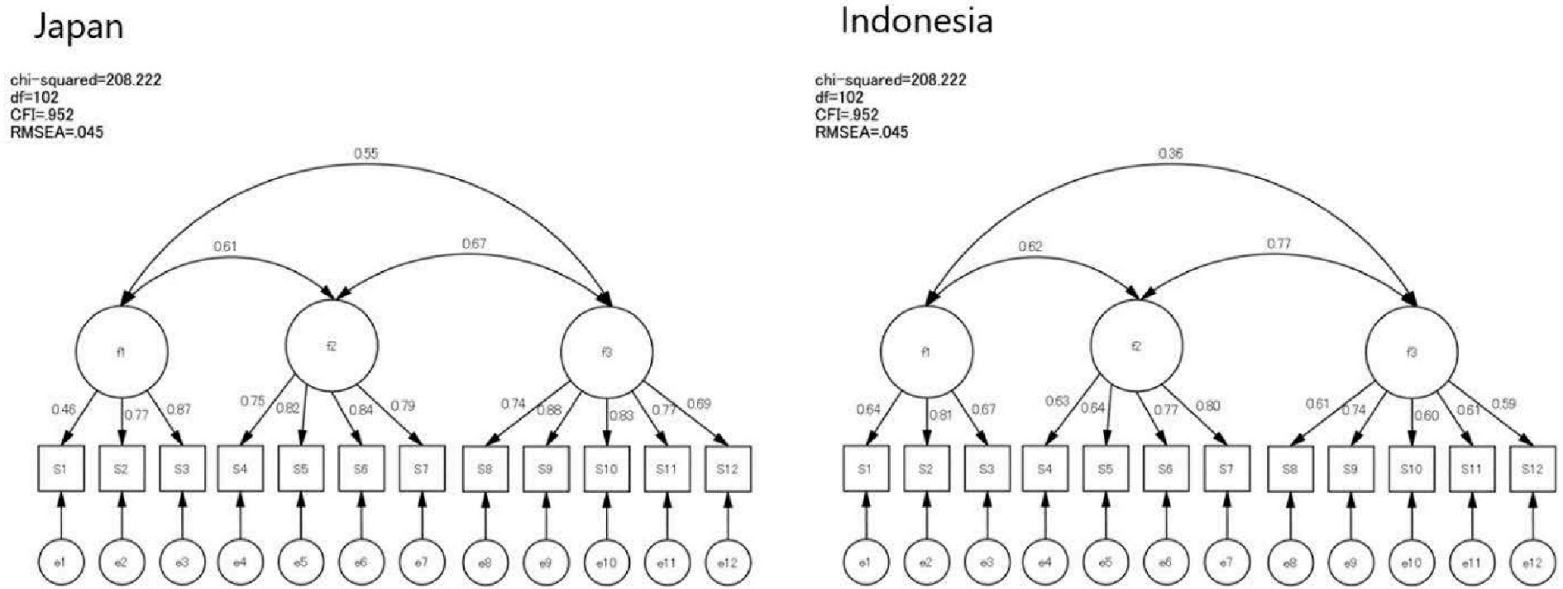
Factor analysis with no constraints.

**Figure 4.**
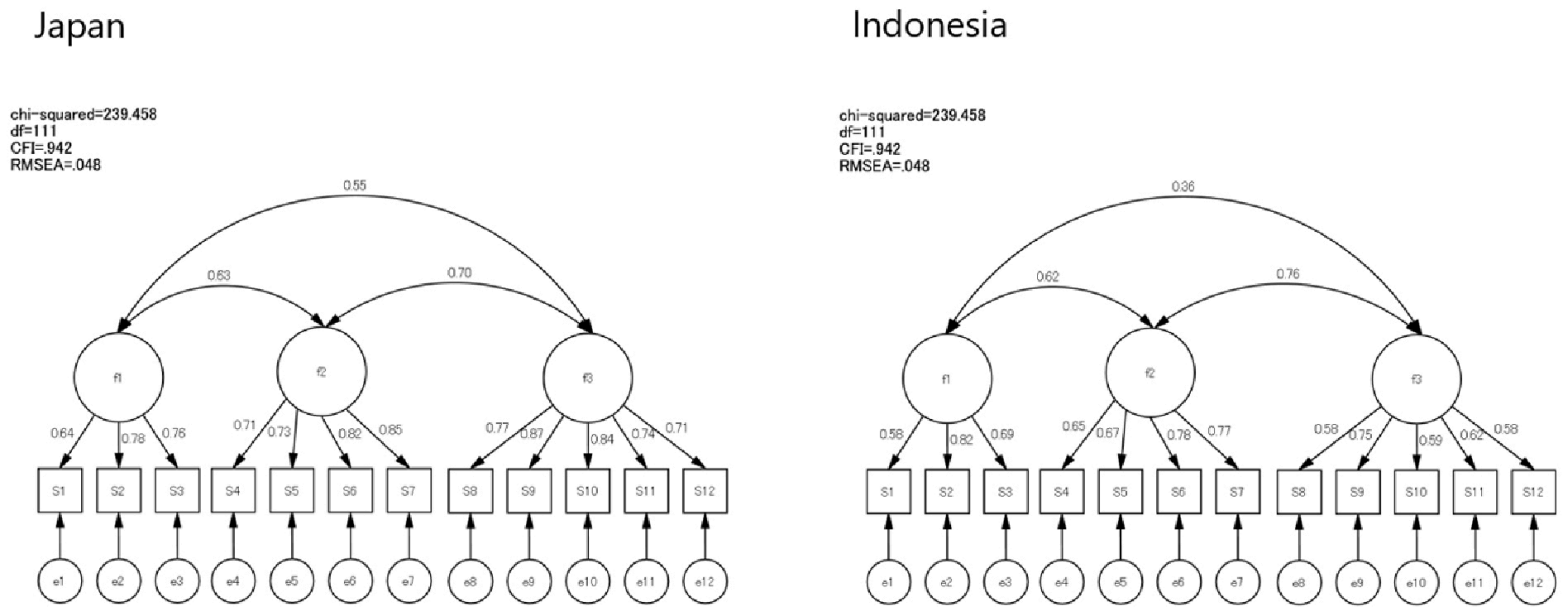
Full metric invariance.

**Figure 5.**
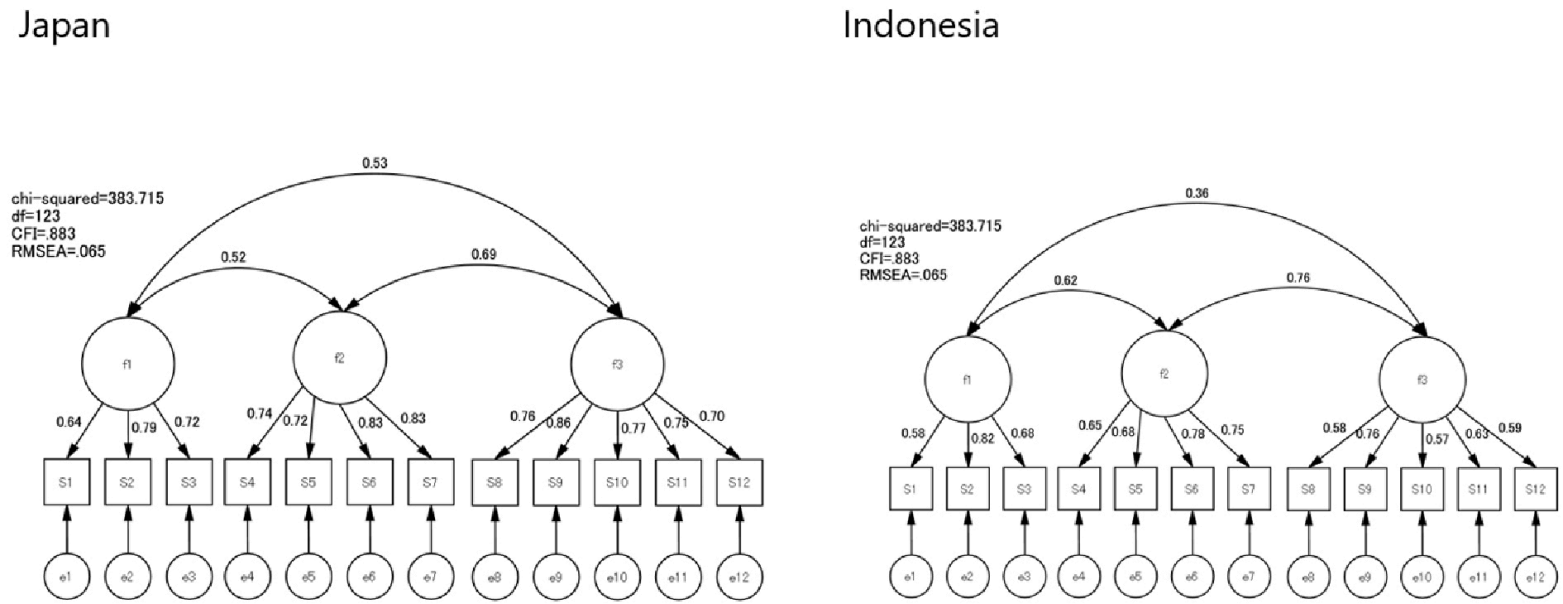
Scalar invariance.

To evaluate the cross-cultural validity of the e-HLS, a Multi-Group CFA was conducted. In this analysis, data from both countries were incorporated into the same dataset. First, a factor analysis without constraints was performed to confirm the consistency of the factor structure. This analysis yielded results with high goodness of fit (Figure 3). Next, full metric invariance was tested to verify the equivalence of factor loadings (Figure 4). The results showed high model fit, indicating consistency in factor loadings for each item across both countries. Furthermore, when constraints were strengthened to test scalar invariance (Figure 5), the model fit decreased, and full scalar invariance was not confirmed. The comparison is presented in Table 5. These findings revealed that although full metric invariance was supported, scalar invariance was not supported.

**Table 5.** Comparison of the Results of the Multigroup Confirmatory Factor Analysis.

	RMSEA	CFI	SRMR	TLI	df	Chi-squared	AIC
No restrictions	0.045	0.952	0.055	0.928	102	208.222	364.222
Full metric invariance	0.048	0.942	0.069	0.932	111	239.458	377.458
	△0.003	△0.010	▲0.014				
Scalar invariance	0.065	0.883	0.075	0.875	123	383.715	497.715
	△0.020	△0.069	▲0.020				

## Discussion

### Findings summary

This study aimed to develop Japanese and Indonesian versions of the e-HLS, validate their reliability and validity, and assess their intercultural validity, thereby expanding their potential for international comparisons. The content validity, construct validity (structural, cross-cultural, convergent, and discriminant validity), and reliability (internal consistency) of the Japanese and Indonesian versions of the e-HLS were confirmed.

### Characteristics of the participants

This study involved undergraduate nursing students from Japan and Indonesia. The original e-HLS was intended for college students, and its reliability and validity were assessed with a representative sample of Taiwanese college students from a wide range of faculties ^(15)^. Medical students reported higher e-HLS scores on all three factors ^(13)^. Moreover, more than 90% of the participants were female in both countries, as the study only included nursing students. The e-HLS scores have been reported to show gender differences ^(15)^. Therefore, the e-HLS scores of the participants in this study were possibly high owing to the large proportion of female participants, and it is difficult to discuss the distribution of the scores in each country based on the present results. However, we believe that the nursing students included in this study encountered health information frequently through classes and practical training and were an appropriate target population for examining the validity and reliability of the e-HLS for young people in both countries. Furthermore, no item in the e-HLS showed a ceiling effect, and the subsequent analysis was considered reliable.

### Validity

This study confirmed scale validity according to the COSMIN guidelines. For content validity, the original conceptual framework and forward-translated questionnaire items were reviewed by two researchers, including the authors, for the Japanese version, and by four researchers for the Indonesian version. Additionally, a pilot survey was conducted to assess the clarity and difficulty of the items and ensure content validity.

Regarding construct validity, CFA was used with fit indices such as CFI, RMSEA, GFI, and AGFI as criteria for acceptable fit; the analysis confirmed the validity of the structural aspect as per the original structure, thus ensuring construct validity ^(24), (25)^. The cross-cultural validity confirmed the configural and full metric invariance. Configural invariance, along with high goodness of fit, indicated the potential equivalence of the factor structures between the two countries. Full metric invariance confirmed that the factor loadings were the same across both countries, suggesting that the e-HLS can be applied to different cultural backgrounds. However, scalar invariance was not supported. Scalar invariance requires constraints on the thresholds between items and covariances between factors. The failure to confirm this suggests that the intercepts for e-HLS Items vary across countries. This highlights the importance of appropriate analytical methods and constraint applications when evaluating cross-cultural validity and comparing data across countries.

The strength of the e-HLS is its factor structure, which complies with Nutbeam’s framework ^(2)^, and each factor has been reported to be individually related to health behaviors and other factors. Previous studies using the e-HLS reported that critical and interactive eHealth literacy was related to health services utilization ^(4)^. In a study designed to examine the associations among various individual factors, eHealth literacy, and health-promoting lifestyles, participants who majored in medical fields showed greater concern regarding their health and frequently sought health information, exhibited better eHealth literacy, and followed a positive health-promoting lifestyle. Furthermore, the study indicated that college students with higher critical eHealth literacy engaged more in health-promoting activities than those with functional and interactive literacy ^(26)^. The fact that the Japanese and Indonesian versions of the e-HLS have the same factor structure as the original version and a certain degree of cross-cultural validity is the most significant finding of this study, suggesting the possibility of conducting international comparisons in the future.

### Reliability

Cronbach’s alpha for each subscale and overall scale satisfied the criterion. Additionally, each subscale demonstrated a high factor loading, indicating that each item was capable of explaining its respective subscale. Therefore, the Japanese and Indonesian versions of the e-HLS achieved satisfactory reliability.

### Discriminative validity

The results of discriminative validity revealed that functional and interactive eHealth literacy increased significantly with the progress of students’ years of study; however, critical eHealth literacy did not increase. Thus, the hypothesis was partially verified. The finding that critical literacy did not improve over the course of undergraduate study suggests the need for measures for improvement. Educational opportunities to improve eHealth literacy range from unstructured offerings on social media (e.g., Instagram) to educational videos by subject matter experts (e.g., YouTube) to comprehensive eLearning offerings (e.g., Massive Open Online Courses) ^(27)^. In the future, interventions involving these educational components should be implemented, and the e-HLS should be used as an outcome measure to assess intervention effects.

### Limitations and future directions

This study has some limitations. First, this was a cross-sectional survey administered only to students from one nursing faculty at one university each in Japan and Indonesia. The limited number of universities and faculties and the small amount of data from male participants owing to the characteristics of nursing faculties reduces the generalizability of these findings. While the cross-cultural validity of the e-HLS was confirmed, whether the same results would be obtained if other institutions and faculties were included in the study remains to be confirmed.

Second, there was a difference in the response rates between Japan and Indonesia. This might be attributed to the fact that, in Japan, all classes transitioned online due to the COVID-19 pandemic, potentially leading to survey requests being overlooked amidst the sudden receipt of innumerable emails. Additionally, the pandemic made it difficult for researchers to approach participants directly because of restricted access to institutions such as universities and research facilities. Social changes and anxiety related to the pandemic may have also contributed to the low response rate. This limits the generalizability of this study regarding eHealth literacy among young people.

Third, the e-HLS is an existing validated scale with an established factor structure, and therefore, EFA was not conducted in this study. Instead, CFA was employed to assess the fit of the predefined factor structure. However, conducting EFA with a pilot sample could have provided further insights into potential cultural differences, and it is acknowledged as an important consideration for future research.

Finally, this study followed the COSMIN guidelines and confirmed the content validity, construct validity, and reliability of the e-HLS. However, test-retest reliability, measurement error, and responsiveness were not evaluated, which are important aspects for assessing the scale’s reliability. Furthermore, reproducibility, comorbidity, and predictive validity were not confirmed in this study. These areas should be addressed in future research to provide a more complete evaluation of the scale’s psychometric properties.

Despite these limitations and challenges, the development of the Japanese and Indonesian versions of the e-HLS, along with their verified reliability and validity, can contribute substantially to the assessment of eHealth literacy among young individuals. Given the constantly changing nature of the information available on the Internet, this scale can play an important role in evaluating whether the youth obtain accurate information from reliable sources to maintain their health. Further empirical research using this scale is necessary.

### Conclusion

This study focused on eHealth literacy assessed by the e-HLS. Japanese and Indonesian versions of the e-HLS were developed and their reliability and validity were confirmed. The e-HLS consists of three subscales that assess the ability to understand, access information, and appraise health-related information. Given their established validity and reliability, the Japanese and Indonesian versions of the e-HLS are suitable for use as measures comprising the same three subscales as the original version.

## Article Information

### Conflicts of Interest

None

### Acknowledgement

We are grateful to the Global Cooperation Institute for Sustainable Cities (GCI) at Yokohama City University for their support throughout this study. We also sincerely thank all the students who participated in the survey for their valuable time and contributions.

### Author Contributions

Yayoi Shoji: Data Analysis, Review & Editing, Manuscript Writing, Study Design, Data Interpretation, Statistical Analysis, Data Collection, Methodology. Andi Masyitha Irwan: Conceptualization, Data Collection. Ryota Ochiai: Data Analysis, Review & Editing, Manuscript Writing, Study Design, Supervision, Data Interpretation, Statistical Analysis, Data Collection, Methodology. Syahrul Syahrul: Conceptualization, Data Collection. Eriko Shinohara: Conceptualization, Data Collection. Andi Muhammad Fiqri: Conceptualization, Data Collection. Shoko Takeuchi: Conceptualization, Data Collection. Erfina Erfina: Conceptualization, Data Collection. Mariko Iida: Project Administration, Validation. Ariyanti Saleh: Conceptualization, Data Collection. Fusae Moriguchi: Conceptualization, Data Collection. Sachiyo Nakamura: Conceptualization, Data Collection. Yuka Kanoya: Conceptualization, Data Collection.

### Approval by Institutional Review Board (IRB)

This study was approved by the Ethics Committees of Yokohama City University (Approval number: A210100029, Approval date: February 2, 2021) and Hasanuddin University (Approval number: UH23010022, Approval date: January 11, 2023).

## Supplement

Supplementary Material 1

Supplementary Material 2

Supplementary Material 3
