# Supplementary material for: Development of Japanese and Indonesian Versions of the electronic-Health Literacy Scale
Source: JMA J. 2025 Aug 8;8(4):1153–64. doi: 10.31662/jmaj.2024-0282 (PMC12598211; doi:10.31662/jmaj.2024-0282)
Supplement: Supplementary Material 1 [file 2433-3298-8-4-1153-s001.pdf]

eHealth Literacy Scale

Level	item
functional	1. I cannot understand the symbols (such as BMI, Body Mass Index) and wording about health information.<br>2. I find the online health information difficult to understand.<br>3. I find the mathematical formulas provided in online health information difficult to calculate. (e.g., the algorithm of calorie consumption, BMI).
interactive	4. I can locate health information efficiently through search engines.<br>5. I pay attention to and obtain new knowledge about online health information.<br>6. I know how to get what I need from online health information.<br>7. I understand the online health information I have obtained.
critical	8. I will think about whether the online health information applies to my situation.<br>9. I try to find different sources to verify the credibility of health information.<br>10. I evaluate the validity and reliability of online health information.<br>11. I will browse various discussions and make a decision or action that is good for health.<br>12.When I have questions or doubts about online health information, I use other channels to verify the information.
