# Supplementary material for: Development of Japanese and Indonesian Versions of the electronic-Health Literacy Scale
Source: JMA J. 2025 Aug 8;8(4):1153–64. doi: 10.31662/jmaj.2024-0282 (PMC12598211; doi:10.31662/jmaj.2024-0282)
Supplement: Supplementary Material 2 [file 2433-3298-8-4-1153-s002.pdf]

Indonesian Version of e-Health Literacy Scale (e-HLS)

1. Sangat Tidak Setuju
2. Tidak Setuju
3. Netral/Ragu-ragu
4. Setuju
5. Sangat Setuju Sekali

1	Saya tidak mengerti singkatan atau kata-kata tentang informasi kesehatan (IMT, indeks massa tubuh, dll.).
2	Sulit untuk memahami informasi kesehatan di internet.
3	Sulit untuk menghitung rumus perhitungan kesehatan yang tersedia di internet (contoh: menghitung jumlah asupan kalori, BMI, dll)
4	Saya dapat menemukan informasi kesehatan secara efisien dengan mesin pencari.
5	Saya memperhatikan informasi kesehatan di internet untuk mendapatkan pengetahuan baru
6	Saya tahu bagaimana cara mendapatkan informasi kesehatan di internet
7	Saya dapat memahami informasi kesehatan di internet yang saya peroleh
8	saya memikirkan apakah informasi kesehatan yang ditemukan di internet sesuai dengan kondisi saya
9	Untuk memastikan keabsahan dari informasi kesehatan, saya mencoba memeriksa berbagai sumber informasi
10	Saya bisa menentukan keabsahan dan keandalan sumber informasi kesehatan dari internet.
11	Saya mengambil keputusan dan tindakan terkait kesehatan setelah menganalisis berbagai diskusi
12	Jika saya memiliki pertanyaan atau ragu tentang informasi kesehatan di internet, saya akan menggunakan cara lain untuk memastikan informasi tersebut
