# Supplementary material for: Development of Japanese and Indonesian Versions of the electronic-Health Literacy Scale
Source: JMA J. 2025 Aug 8;8(4):1153–64. doi: 10.31662/jmaj.2024-0282 (PMC12598211; doi:10.31662/jmaj.2024-0282)
Supplement: Supplementary Material 3 [file 2433-3298-8-4-1153-s003.pdf]

Japanese Version of e-Health Literacy Scale (e-HLS)

1. 全くあてはまらない
2. あまりあてはまらない
3. ややあてはまる
4. かなりあてはまる
5. 非常にあてはまる

1	健康情報に関する略語（BMI＝ボディマス・インデックスなど）や言い回しを理解できない。
2	インターネット上の健康情報を理解するのが難しい。
3	インターネット上の健康情報のなかで出てくる数式を計算するのは難しい（消費カロリーの計算や BMI など）。
4	検索エンジンで健康情報を効率よく見つけることができる。
5	インターネット上の健康情報について注意を払い、新たな知識を得ている。
6	インターネット上の健康情報から自分に必要な情報を得る方法を知っている。
7	自分が得たインターネット上の健康情報を理解できる。
8	インターネット上の健康情報が自分の状況に当てはまるかどうかを考えるようにしている。
9	健康情報の信憑性を確認するため、複数の情報源にあたるようにしている。
10	インターネット上の健康情報の妥当性と信頼性を見極めるようにしている。
11	様々な議論に目を通したうえで健康によい選択や行動を決めるようにしている。
12	インターネット上の健康情報について疑問や疑念があるときは、別のルートを用いて情報の正しさを確かめている。
